# Understanding the futility of countries’ obligations for health rights: realising justice for the global poor

**DOI:** 10.1186/s12914-016-0090-2

**Published:** 2016-06-03

**Authors:** John Barugahare, Reidar K. Lie

**Affiliations:** Department of Philosophy, Makerere University, Kampala, Uganda; Department of Philosophy, University of Bergen, Bergen, Norway

**Keywords:** Health opportunities, Marginalised populations, Right to health, Global distributive justice, Obligations, Low Income Countries, Developing countries

## Abstract

**Background:**

Although health is a right of all individuals without any distinction, the realisation of this right has remained very difficult for the marginalised populations of poor countries. Inequitable distribution of health opportunities globally is a major factor in explaining why this is the case. Whereas the Protection, Promotion and Fulfilment of the health rights of poor country citizens are a joint responsibility of both domestic and external governments, most governments flout their obligations. So far disproportionate effort has been dedicated to reaffirming and interpreting these obligations as opposed to investigating the fundamental question regarding why these obligations have nevertheless remained largely unfulfilled. Further the normative question regarding what ought to be done about the shortcomings of current obligations has been largely ignored.

**Methods:**

We conduct a critical content analysis of existing literature on efforts towards the realisation of the health rights of marginalised populations in our attempt to ascertain their capacity to guarantee basic health opportunities to marginalised populations. In our analysis we treat issues of ‘health rights’ and ‘justice in global health’ as having unity of purpose – guaranteeing basic health opportunities to the marginalised populations.

**Results:**

We identify two sets of reasons for the failure of present obligations for global distributive justice in general: a set of ‘superficial reasons’ and a set of ‘fundamental reasons’ which account for the superficial reasons.

**Discussion:**

In order to overcome these reasons we propose a strategy which consists in specifying a number of minimum and less-demanding obligations for both external and domestic governments to guarantee to all individuals a certain threshold of health goods and services. We argue that these minimum obligations can be freely accepted and fully complied with or enforced with “a thin system of enforcement” without significant threat to national sovereignty and autonomy.

**Conclusion:**

The futility of countries’ obligations for the health rights of the global poor as is the case with global distributive injustice is because of lack of political will to specify and enforce such obligations. Minimum obligations should be specified and enforced with a “thin system” which is consistent with principles of national sovereignty and autonomy.

## Background

Although health is a right of all individuals without any distinction its realisation has remained extremely problematic for most citizens of poor countries. Inequitable distribution of health opportunities in form of health resources (and other social determinants of health) both within and between countries is one of the major impediments to the realisation of the health rights of the globally marginalised populations. Given the close connection between fairness in the distribution of health resources and the realisation of health rights for all, we place our discussion in the broader context of global distributive justice and countries’ domestic and extraterritorial obligations to ensure justice in the distribution of health opportunities. On the basis of principles 55 and 56 of the UN Charter [1]; the wide recognition of Official Development Assistance (ODA) target of 0.7 of GNP [2]; the nature and scope of countries’ obligations for the realisation of Economic, Social and Cultural Rights (ESCR) as interpreted by The Limburg principles [3] especially paragraph 26, our discussion naturally covers both domestic and extraterritorial obligations for global distributive justice which are for the most part framed as human rights obligations. Our point of departure is a key note that despite the current wide political recognition of countries’ joint responsibility to ensure global distributive justice of which health is one dimension, most countries do not fully comply with their obligations and consequently global distributive injustice has continued to deepen. Whereas this fact and its consequences for the health rights of Low Income Country (LIC) citizens are well known, the hard question regarding why most countries do not fully comply with their obligations has been largely avoided. This paper examines the reasons why, to a larger extent, most countries’ obligations have remained futile. After accounting for this futility, we go ahead to propose what ought to be done about this situation.

In this analysis our major concern is with the dimension of global justice which has been labeled “global social justice” by the United Nations (UN) [4], and more specifically described as “global distributive justice” by Charles Beitz [5]. Our interest is in how the latter affects the realisation of the health rights of marginalised populations of developing countries. Our discussion is based on the thinking that “social justice derives from equality of rights for all peoples and the possibility for all human beings, without discrimination, to benefit from the economic and social progress disseminated and secured through international cooperation” (emphasis added) [4]. Our discussion is also based on the UN’s recognition that “[…] The international human rights system is an important way of advocating and enforcing fairer distribution of resources in the world” [4]. Further, the discussion demonstrates that the reverse of the UN’s view is also true; that is, a fairer distribution of global health opportunities especially health resources, will go a long way towards guaranteeing basic health rights to marginalised populations. Hence, we are here treating human rights obligations and obligations defended in philosophical debates about global distributive justice as flip sides of a coin in the pursuit of global distributive justice and/or the realisation of the health rights of the global poor. We shall provide an account for the failure of countries’ obligations at two levels: the first level consists of reasons that can be regarded as ‘superficial’. These reasons arise from the lack of precision and rigour in current obligations as well as their lack of an enforcement mechanism. The second level of our account is a set of what we regard as ‘fundamental reasons’ which account for the superficial reasons. The analysis ends with a normative case for a piecemeal strategy that can ensure an incremental achievement of justice in the global distribution of health opportunities thereby leading to consistent progress in the realisation of the health rights of marginalised populations. Central to the strategy we propose the adoption of Martha Nussbaum’s “minimal conception of social justice” [6] as key to understanding the requirements and the feasibility of global distributive justice. In particular the extrapolation of Nussbaum’s concept of the “minimum” is more relevant in discussions of the right to health in light of the concept of “Core obligations” of States Parties outlined in paragraphs 43–45 of the International Covenant on Economic, Social and Cultural Rights (ICESCR) General Comment number 14.

### Preliminaries

Since our discussion is grounded in global distributive justice and the basic rights of marginalised populations, we have chosen to focus on the rights of the global poor who suffer most from current global resource maldistribution and hence cannot realise their right to basic health services. In our analysis we will not be concerned with the question of whether there are morally binding transnational obligations for distributive justice. This is a crucial background question for the kind of analysis we are engaged in. Although there still persists some theoretical controversy on the legitimacy of stringent transnational obligations of distributive justice, we treat this question as settled with an affirmative answer. We base our enquiry on the implied acceptance of these obligations by countries as reflected in their various political commitments, such as Official Development Assistance (ODA), ratification of the relevant human rights instruments among others. Our view is that these political commitments can be interpreted as promises made by countries to implement and fulfil obligations suggested by those documents and instruments. There are several views that have convincingly demonstrated that such promises give rise to morally binding obligations on the promisor because they confer (moral) rights to the promisee [7–9]. Generally there are a number of financial and other kinds of promises by developed countries to developing countries in the spirit of international cooperation with a view of ensuring global distributive justice as expressed in various ways [10–12]. However, despite these political commitments global distributive injustice seems to deepen even further and it is this situation that leads us to the question of why this is the case. We need to emphasise that the futility of countries’ obligations refers to both domestic and extraterritorial obligations of countries. With regard to domestic obligations, emphasis is on obligations of resource poor countries (or all countries that claim a share of international health resources). Apart from health financing which is treated as a special case in our current discussion, there are other poor country obligations such as equitable distribution of available health resources, implementing evidence-based health policies and programs and all those obligations suggested by the concept of ‘governments and ministries of health as stewards’ of health sectors.

To illustrate briefly the extent of the futility of extraterritorial obligations, for example, we can turn to a summary of countries’ compliance with ODA targets. With respect to High Income Countries (HICs), most of them have largely not fully fulfilled their obligations emanating from ODA promises. In its 1969 Report, the Pearson Commission recommended in reference to HICs that “… Public or government assistance in the form of grants, or low or interest-free loans should make up 0.70 % of the gross national product by 1975 and in no case later than 1980 [12]. The UN General Assembly adopted a Resolution confirming this target as the minimum of ODA as early as 1970 [2]. Although this target was initially controversial in the opinion of some countries, it eventually gained wider acceptance by a majority of member countries [2]. Whereas the acceptance of this target constituted a promise to the developing countries, very few developed countries that accepted this target have ever reached it. By 2009 the highest ever registered ODA as an average percentage of Gross National Incomes (GNIs) of the Organisation for Economic Co-Operation and Development (OECD) Development Assistance Committee (DAC) was 0.33 % in 2005 [13], while the lowest went as low as 0.22 % in 1997 [14]. The consequence of this situation has been shown to be the continuously deepening global inequalities and inequities in the global distribution of wealth, health as well as general quality of life [15]. This extent of futility of countries’ obligations for global distributive justice partly explains the persisting difficulty in fulfilling the health rights of developing country citizens and other marginalised populations.

## Results

Even though there are many categories of human rights obligations under the two major categories - so-categorised as positive and negative obligations - our discussion is primarily concerned with the ‘positive obligations’ and in particular the joint obligation of all countries to contribute health resources sufficient to guarantee a certain minimum level of health opportunities to all individuals globally. We emphasise the concepts of ‘guaranteeing’ and the ‘minimum’ as key in demonstrating the futility of current obligations. In this regard, our account for the futility of current obligations consists in identifying reasons why existing obligations and their various interpretations do not, in practice; and cannot, in principle, guarantee to the global poor access to a certain minimum level of health goods and services and why it is extremely difficult to judge States Parties as having made negligible or no effort towards that end.

## Reasons for the futility of current obligations

Our critical examination of the various attempts to explain the futility of current obligations of countries for global distributive justice or fulfilment of Economic, Social and Cultural Rights (ECSR) [16, 17] reveals two levels or categories of reasons for the futility of countries’ obligations. The first category is sieved directly from the manner current obligations are framed. We call these reasons ‘superficial reasons’. However, a deeper investigation reveals that there are other reasons which explain the existence of these ‘superficial reasons’. We call these deeper reasons ‘fundamental reasons’. Our view is that without identifying these fundamental reasons the futility of countries’ obligations remains inexplicable. For that matter if we are to successfully work around the current futility of countries’ obligations we need a solution to these fundamental reasons. With regard to superficial reasons one finds that by way of their phrasing and various attempts at interpreting them, current obligations are deficient in precision and rigour regarding actions that both domestic and external governments must take to ensure the realisation of global distributive justice and consequently the health rights of LIC citizens (and other marginalised populations). The second superficial reason is the absence of an enforcement mechanism for these obligations. With regard to fundamental reasons, we offer two explanations: lack of political will to specify and enforce morally binding obligations of global distributive justice and pessimism about the desirability and feasibility of a coercive global enforcement mechanism for these obligations.

### Superficial reasons for the futility of current obligations

In our examination of the two superficial reasons mentioned above, we shall take lack of enforcement as self-evident and at the same time treat its consequence for countries’ compliance with their obligations as self-explanatory. That is to say, lack of any perceived threat in case of non-compliance with these obligations provides a strong temptation for governments to do as little as they wish or nothing at all with regard to their obligations, particularly domestic resource allocations (in the case of Low and Middle Income Countries (LMICs)) and international resource transfers (in the case of High Income Countries (HICs)). Therefore, a detailed analysis of superficial reasons will be on illustrating how vagueness and lack of rigour in current obligations make it easy for most national governments to defy their obligations. Further we argue that lack of any perceived threat in cases of non-compliance, is what makes this breach quite tempting and the lack of specific and objective grounds for holding countries morally blameworthy if they flout their obligations.

To begin with, an examination of obligations of HICs relating to health financing in LMICs reveals that the promises embedded in acts of countries’ ratifying the various human rights instruments are vague. This vagueness denies the purported obligations the rigour expected of obligations of justice. In the first place current obligations are characterised by a number of exception clauses which leave them open for interpretation as to what countries are morally required to do in respect of the health rights of people outside their countries. This challenge makes it difficult to objectively judge any external governments as morally guilty of not fulfilling its obligations whenever they either do too little or nothing at all to ensure that individuals outside their countries realise their basic health rights.

With regard to constrained rigour of current obligations it is important to see how they have been interpreted. External obligations (to assist) have been interpreted as follows:

For the avoidance of any doubt, the Committee wishes to emphasize that it is particularly incumbent on States Parties and other actors in a position to assist, to provide international assistance and cooperation, especially economic and technical which enable developing countries to fulfil their core and other obligations (emphasis added) [18].

Whereas the use of expression “incumbent on” seems to make this obligation rigorous enough, its stringency is severely diminished by an addition of a proviso “those in a position to assist”, yet, without going ahead to specify how, in case of non-compliance, it could be objectively determined whether a given State Party was in position to assist or not. This means that even if there were an enforcement mechanism for this obligation it would be practically impossible to objectively claim that a State Party has refused to honour its obligation. Therefore, it can be inferred that if “obligations to assist” are understood as obligations of justice which are meant to be stringent (and potentially enforceable), then the current human rights discourse does not effectively impose on HIC governments any obligations of justice which can guarantee to citizens of developing countries the realisation of their right to basic health opportunities. That is, given the framing of current obligations to assist, external actors bear no stringent obligation relating to international health resource transfers to LIMCs and this partly explains why most of these obligations have remained futile.

It is not only the HICs that have flouted their obligations. Most LMIC governments can be implicated in what may count as injustice in health care and a violation of health rights of their own citizens. The futility of some of LIC governments’ obligations particularly human rights obligations to their citizens can also be attributed to vagueness and lack of rigour. As we mentioned earlier, even though LMIC government obligations are numerous, we shall here concentrate on health financing in order to demonstrate our argument.

In the case of countries’ obligations to Promote, Protect and Fulfill Economic, Social and Cultural Rights particularly the right to health [19], Article 2 (1) of the ICESCR obligates each State Party to take the necessary steps “to the maximum of its available resources” to the realisation of these rights (emphasis added). This has been interpreted to mean that:

In order for a State Party to be able to attribute its failure to meet at least its minimum core obligations to a lack of available resources it must demonstrate that every effort has been made to use all resources that are at its disposition in an effort to satisfy, as a matter of priority, those minimum obligations [20].

In addition the World Health Organisation (WHO) has emphasised that in determining the violations of the right to health “it is important to distinguish the inability from unwillingness of a State Party to comply with its obligations under article 12 [of the ICESCR]” [21]. A further proviso is that “If resource constraints render it impossible for a State Party to comply fully with its Covenant obligations, it has the burden of justifying that every effort has nevertheless been made to use all available resources at its disposal […] (emphasis added) [22]. This interpretation is reproduced in the Maastricht Guidelines on violations of ESCR (par. 13) [23], where the burden of proof of inability falls on the State Party. It is important to bear in mind that this clause also applies to HIC government obligations to assist. Further, regarding the determination of violations of obligation to fulfil, it has been interpreted that violations of obligation to fulfil occur through, among other things, a state’s “insufficient expenditure or misallocation of public resources; […]” (emphasis added) [22]. What is crucial to note is that the essence of providing these various interpretations is that they should be neither vague nor ambiguous as to what they require of State Parties with a hope that such clarity will induce wide compliance. However, these obligations as interpreted in General Comment 14 of ICESCR and other Principles and Guidelines ([3, 23] still fail to overcome their vagueness, plasticity and consequently some of these interpretations have instead produced contradictions as shown below.

Whereas at first reading the above statements of obligations seem precise and rigorous enough, a critical examination of these phrases reveals serious problems with regards to their precision and rigour. In the first place, how much, or what percentage, of a country’s resources (GDP or annual budget) allocated to health shall be accepted as the maximum of resources available at its disposal to fulfil the right to health? On this fundamental issue of “to the maximum of its available resources”, paragraph 26 of the Limburg Principles clarifies that “Its available resources refers to resources within a state and those available from the international community through international co-operation and assistance”. However, still there is no suggestion as to how much of such external resources each LIC is entitled to or an officially recognised mechanism of how to determine such amounts of resources. We have proposed, defended and demonstrated such a mechanism elsewhere [24]. The current discretion regarding health resource allocations is directly implied by paragraph 71 of the Limburg Principles. The limit placed on the ‘margin of discretion’ given to States Parties in the above-mentioned paragraph (in determining violations) is indeterminate; this limit is a sort of thing about which reasonable people can disagree. For example, in Uganda, whereas there has been wide public and expert protest against the government’s neglect of the health sector in budget allocations, bearing in mind that the Abuja Declaration like all Declarations is not legally binding to the extent that its implementation depends purely on political will, there is no stringent and potentially enforceable minimum percentage of national budgets that must be allocated to health” [25–28]. Further, even though the WHO has observed and recommended that all health-aid recipient countries (in the WHO Africa region) need to increase percentages of their domestically generated annual budget resources to health, there is no specific level recommended [29]. Therefore, current ‘obligations’ of countries do not place any country under any obligation to allocate a certain minimum of their financial resources to the health of marginalised people and this partly explains why it has remained very difficult for citizens of LICs and in some MICs to realise their basic health rights.

Further, instead of clarifying obligations, some efforts at interpreting these obligations have produced contradictions with some of the key principles for implementation of these obligations. One glaring contradiction pertains to the essence of distinguishing between inability and unwillingness to fulfill obligations. By making reference to paragraphs 25 – 28 of the Limburg Principles, Paragraphs 9 and 10 of the Maastricht Guidelines on violations categorically exclude ‘lack of resources’ as a valid excuse for failure of a State Party to fulfil its obligations. So, if the essence of determining violations is either that a State Party be reprimanded or be made to suffer sanctions, how morally justified would the international community be in reprimanding or punishing a State Party which is, because of resource scarcity, unable rather than unwilling, to fulfil its core minimum obligations ? Is the negation of ‘resource scarcity’ as an excuse limited to determining violations relating to obligations of ‘non-discrimination’ and ‘equity in resource allocation’ and the like? This specification would make better sense but unfortunately in the available literature there is nothing to suggest this.

Secondly, regarding the need to distinguish inability from unwillingness of States Parties to comply with their obligations, this requirement calls for precision in judging countries on their performances in order to be able to objectively say that country ‘A’ is able but unwilling or country ‘B’ is willing but unable to fulfill their obligations. The required precision in making this distinction presumes the existence of a mechanism by which to make this precise distinction, yet, there is no officially recognised mechanism of such kind. Therefore, with regard to resource allocations it is technically impossible to objectively judge any country as unwilling or unable to comply with its obligations. Hence, the seeming stringency of this obligation again vanishes with the impossibility of objectively judging any government as either unwilling or unable to guarantee certain levels of health opportunities to its citizens (in the case of LMICs).

In the case of HICs, it becomes impossible to objectively claim that a country has flouted its obligation by transferring low or no amounts of health resources to LMICs. The question here would be: ‘how much is such a State Party morally required (or legally obliged) to transfer to LICs, and/or particularly to which LIC, and how should it be determined’? In the existing literature, particularly literature on obligations and their various interpretations (with exception of ODA targets), it is extremely difficult, if possible at all, to find an answer to this question. The consequence of this is that lack of an impartial basis for assigning moral blame to governments for their unwillingness to ensure a just level of health opportunities for marginalised individuals globally leads to complacence among most governments. Even if the ODA target were to be accepted as the actual size of positive extraterritorial obligations, the present lack of coordination in international resource transfers implies that still inequitable distribution of global health resources would persist along with its consequence for the realisation of the health rights of most LIC citizens. Again, even if there were to be an enforcement mechanism for this obligation, this lack of precision as to how to distinguish between countries’ inabilities and unwillingness to comply with their obligation to assist would make it extremely difficult to hold any government accountable.

Another attempt at précising governments’ obligations to ensure the realisation of the right to health by guaranteeing a certain minimum level of health opportunities to all individuals has been through the definition of “Core obligations” of States Parties as listed in the ICESCR, General Comment 14, Paragraphs 43 to 45. But still these core obligations in general simply specify what types of services individuals must have access to while remaining noncommittal on how much resources or what percentage of national budget (or even what percentage of external resources) ought to be committed to such services. The “minimum core obligations” as interpreted by Maastricht Guidelines on violations still do not define the minimum in, for example, Nussbaum’s terms of “some appropriate threshold level” in her idea of the “minimal account of social justice” [6]. This kind of minimum which makes reference to a specific threshold would be a determinate level of, for example, a minimum global health-resource per capita which must be guaranteed to every individual through fulfilment of domestic and extraterritorial obligations as we demonstrated elsewhere [24].

What is noteworthy about core obligations is the addition of “the right to health indicators and benchmarks” [30] as operationalised in specifying targets for health-related Millennium Development Goals (MDGs Four to Six). We can see, of course, how health indicators come close to a definition of a specific threshold, albeit at the level of population health. However, in order to see how these core obligations and health indicators and benchmarks fail to guarantee the necessary precision in holding governments accountable, it is important to look at health indicators in relation to their resource/financial implications.

Bearing in mind that “the right to health does NOT imply the right to being healthy” (emphasis in original) [21], specific health indicators and benchmarks in themselves are neither a sufficient nor necessary criterion for judging a government’s performance on its obligations. This is because the above disclaimer (what the right to health does NOT imply) entails that poor population health outcomes do not constitute conclusive evidence that a State Party has not done the best it can in its specific resource context; nor would it be automatically inferred from improvements in these indicators that a State Party is performing to the best of its efforts in its specific resource context. With reference to the latter case what is implied is that the concept of “progressive realisation” of the right to health (ICESCR Art.1 (2)) towards “the highest attainable standard of health” [31] entails that even if a country has achieved the prescribed health indicators by investing less than the maximum of its available resources, such a country will still be violating the health rights of its citizens because it is unwilling to give them more health opportunities towards the highest attainable standard of health. Even though setting population health indicator targets is very important for health policy and program evaluations and appraisals etc., without specifying the precise level of investment in health for each country especially LICs, the idea of “the right to health indicators” in itself does not help in objectively judging governments’ performance.

Finally, the recognition by the UN Committee on ESCR of extraterritorial obligations relating to ESCR promises hope with regard to countries’ compliance with their obligations. However, the impact of this recognition, especially on international resource transfer, will largely depend on the precision regarding which State Party should transfer how much resources to whom; or on the recognition and implementation of a mechanism such as one we proposed elsewhere [24]. However, this explicit recognition is crucial for the negative obligations of countries and their international companies (not to violate ESCR of citizens where they operate), and for providing clear complaint procedures and mechanisms to those who think their rights have been (positively) violated as reflected in the Committee’s concluding observations on the second periodic report of China [32].

In summary, the above examination reveals that current obligations of countries to guarantee the fulfilment of at least the basic health rights of LIC citizens and thereby ensure justice in global health are not yet obligations in the real sense of obligations of justice which specify moral requirements on the part of obligation bearers and moral rights on the part of the beneficiaries of such obligations. The lack of specificity in current obligations means that there are no objective grounds for judging any State Party as having flouted its positive obligation particularly relating to domestic resource allocation or international transfer. This situation is worsened by the fact that there is no enforcement mechanism for these obligations. But as the analysis above has shown, it would still be pointless to have an enforcement mechanism if it is impossible to objectively judge any country as having flouted its obligations. The ultimate consequence of these two weaknesses is that currently governments perceive no impending threat whenever they contemplate flouting their obligations, whether the threat would be in form of moral blame or a number of sanctions. At best current international resource transfers are treated as charity which falls under the category of duties of humanity rather than obligations of justice and, therefore, cannot guarantee the fulfilment of the relevant basic rights. But as mentioned earlier there are underlying reasons which explain lack of precision and rigour in current obligations as well as lack of any enforcement mechanism in case of non-compliance with these obligations. This is the category of reasons which we have called ‘fundamental reasons’.

### Fundamental reasons for the futility of obligations for global distributive justice

The superficial reasons for the futility of countries’ obligations for justice in global health are not spontaneous but systematic. Our concern in going beyond the superficial reasons is that even though the superficial reasons we have pointed out above are not new and moreover efforts have been made to respond to them through various interpretations of obligations, it remains unclear why these obligations have remained vague and unenforceable. Our major claim here is that at a fundamental level, lack of specificity, rigour and enforcement of these obligations is a symptom of general lack of political will on the part of countries (or State Parties) to specify and enforce these obligations. The second fundamental reason, and specifically a reason for lack of an enforcement mechanism, is general pessimism regarding both the desirability and feasibility of coercive enforcement of these obligations. We later show that these fundamental reasons are not insurmountable and that there are good reasons and possibility to work around them if we want to gain any hope for realising the health rights of marginalised populations.

There is general lack of political will on the part of Sates Parties to specify and enforce obligations of global distributive justice and this directly and negatively affects the realisation of the health rights of marginalised populations in LMICs. The evidence for the general lack of political will to specify actions which countries ought to take as a moral requirement to fulfill their obligations for global distributive justice can be seen in the common theoretical denial of the moral necessity and legitimacy of transnational obligations for distributive justice. In discussions of obligations of countries for global distributive justice there is a widely shared claim that robust principles of distributive justice and their consequent obligations do not go beyond national borders [33–38]. Among these authors Norman Daniels in particular imports this way of thinking into discussions regarding justice in global health. In consideration of the spirited arguments that these authors have put up in defense of this position on the one hand, and the current attitude towards ODA as reflected in the practice of treating international resource transfers as charity on the other, it is not unreasonable to assert that these views have significantly influenced politics and policy, especially among external obligations bearers. So it is very difficult to claim that lack of precision and enforcement mechanism is an oversight, nor is it lack of cognitive capacity to state such obligations in a more specific and stringent manner. Rather it is due to lack of political will that obligations are not framed and treated as real obligations of justice; that is, precise and potentially enforceable. Apart from this theoretical controversy there is more subtle evidence of lack of political will to specify and enforce countries’ obligations for global distributive justice. But before we adduce further evidence we need to emphasise that we have taken it for granted that such obligations exist but what is unclear are reasons why those who believe that such obligations exist have not succeeded in specifying and enforcing such obligations.

Further evidence for lack of political will can be traced from the importance all countries attach to the political values of national sovereignty and autonomy. We treat it as self-evident truth that generally countries are unwilling to give up their freedom in deciding how to manage their domestic affairs such as budget allocations in the case of LIC governments; and how much of their resources they must give out in ODA or any other forms of bilateral or multilateral assistance in the case of HIC governments. In this case the worry is that by specifying what countries must do in terms of domestic budget allocations and international resource transfers with a possibility of enforcing such decisions, countries are robbed of their sovereignty and autonomy in deciding for themselves on these matters. Therefore, our judgement is that the current phrasing – vagueness and constrained rigour – of obligations as well as reluctance to propose and implement an enforcement mechanism is a politically cautious way of going about transnational requirements for global distributive justice.

Therefore, the other fundamental reason for the futility of current obligations is a corollary or an extension of the one above. It has more to do with the desirability, legitimacy and feasibility of enforcement of these obligations against principles of national autonomy and sovereignty. In the view of the UN, for example:

[…] there is an inherent futility in working to achieve greater equality between States in terms of development when there is no authority able to enforce measures that would ensure the realization of such an objective. The United Nations does not possess such authority. International organizations with greater power and influence in economic and financial matters, in particular the World Trade Organization (WTO), the World Bank and the International Monetary Fund (IMF), have different mandates. A world government with an enforceable mandate to ensure equality and justice between its constituents is not on the immediate horizon [4].

This lack of effective and legitimate means to enforce transnational obligations is the major source of pessimism about the feasibility of enforcing obligations of global distributive justice in the same manner as it happens at a national level. But it can as well be said that the required means (institution or agency) for enforcement can be created only if there is general, or at least widely shared, political will to do so. Therefore, for as long as such an enforcement agency does not exist, then it is because there is not yet willingness to create it.

With regard to the fundamental reasons outlined above, lack of political will remains central. Further evidence to this has been alluded to in a more direct way. Such allusions have been made by pointing at serious doubts regarding whether it can be possible to have a truly democratic and fair global institution to do the enforcement. There are some views that there is particularly fear of the threat of imperialism especially against weaker states [37, 39, 40]. Nagel in particular points to a dilemma stemming from ‘the need for effective institutions and the threat of expanding tyranny’. In his view “fortunate nations” fear such developments. “They therefore face the problem of how to create a global order that will have its own legitimacy, but not the kind of legitimacy that undermines strict limits on their responsibilities [for the well-being of non-compatriots]” [37]. In a footnote he adds that “The undemocratic rulers of many poor nations have strong reasons of a different kind to protect their sovereign authority against international encroachment (emphasis added)” [37]. This is an emphasis of the importance all countries attach to national autonomy and sovereignty which, most countries believe, will be eroded to some great extent by specifying and enforcing transnational obligations of distributive justice to which obligations for the health rights of LIC citizens belong. It is these political circumstances that account for the general lack of specificity and rigour in obligations of global distributive justice as well as reluctance to propose and implement an enforcement mechanism which might guarantee wider compliance with these obligations.

Therefore, whereas there is potential for developing mechanisms for specifying and enforcing obligations of countries for global distributive justice, there are deeper reasons why the current obligations are vague and unenforceable. But the question arises: if current obligations are not divested of the conditions that cause their futility, what is the future of global distributive justice? Can the marginalised populations hope to realise even their basic health rights in these circumstances which do not guarantee anything to them? Judging from the current daunting performances of most countries on their obligations and the consequent trend in global distributive justice particularly the global mal-distribution of health opportunities as evidenced by the current trends in global health resource allocation [41, 42], it is obvious that unless the current conditions or reasons which explain the futility of current obligations are circumvented, there is no hope that the marginalised populations will realise their health rights or global justice in general can ever be achieved.

## Discussion

### The way forward

Given our emphasis of the need to guarantee certain minimum health opportunities to marginalised populations, the first crucial step forward is to shift from relying on charity (or perfectly voluntary ODA disbursements), to specific and enforceable obligations of distributive justice among all HIC governments and reject unlimited discretion on LICs regarding health resource allocations in their annual budgets. At a domestic level, such discretion is not compatible with the idea of guaranteeing certain minimum health opportunities. Further still, with regard to ODA the key insight is that perfectly voluntary ODA disbursements or any other forms international health resource transfers that treat such transfers as charity cannot guarantee anything to the marginalised populations. A moral reason for this shift has been offered by Nussbaum in her agreement with Liam Murphy [43]. Nussbaum’s view is that exclusive reliance on voluntary philanthropy has a problem of failing to equitably distribute the burden of alleviating poverty and suffering especially if only a few have to contribute all that is necessary to solve the problem. She rightly observes that “Any system of voluntary philanthropy has this problem”[6]. Another reason is David Hume’s view regarding why we must move away from benevolence to justice. In his view benevolence is inspired by moral impulse and circumstances of unlimited abundance and is therefore unreliable [especially in the current global scarcity of resources] [44]. Lastly we hope that it is possible to agree on certain minimum obligations, enforcement of which can be readily accepted by virtue of the minimal burden they impose on external actors. This is especially possible if it could be demonstrated that these obligations impose a proportionate or fair burden on external actors^1^ and also such obligations are within the resource capacities of individual LICs. We can arrive at such obligations through the adoption of what has been termed “a minimal conception of social justice” [45].

One major and obvious reason for the futility obligations for the health rights of marginalised populations is that all ESCR primarily require resources and yet there is real scarcity of resources. So given this reality it would be a slippery-slope choice to make ESCR justiciable or adopt any measures purporting to guarantee their fulfilment. This excuse can be overcome by the extrapolating Nussbaum’s “minimal conception of social justice” into reasoning about the feasibility of guaranteeing basic health rights for the marginalised populations. The gist of the idea of “a minimal account of social justice” in Nussbaum’s view is that “a society that does not guarantee these [fundamental entitlements/capabilities] to all its citizens, at some appropriate threshold level, falls short of being a fully just society, whatever its level of opulence” [45]. Notwithstanding the ‘possible overstatement of this position with regard to judging a society as unjust’,^2^ particularly due to genuine and severe resource constraints among most developing countries, Nussbaum’s view is still very crucial in understanding the goals and limits of global distributive justice, particularly the idea of basic health goods and services which are supposed to be guaranteed to all individuals as a matter of right. When applied to global distributive justice, “a minimal conception of distributive justice” means that global justice does not aim at achieving a perfectly equal distribution of resources between the global rich and the global poor. Rather, global distributive justice on this account asks for a guarantee to all global citizens “some appropriate threshold level of capabilities” [6]. The concept of the minimum is already widely implied as what is morally required for a just level of material and social well-being – for example, the concept of “minimum core obligations” used in the human rights discourse; the Millennium Declaration, particularly MDGs targets and the WHO’s concept of the minimum health care which targets everybody’s access to an “Essential Health Package” [6] especially in low income countries, among others. The implication we draw from the “minimal conception of social justice” is that external obligations do not have to be as demanding as they are currently thought to be by those who are skeptical about morally binding transnational obligations for global distributive justice. Therefore, the power of the concept of the “minimum” is that these obligations can reasonably be accepted and fulfilled with “a thin system” of enforcement and in some cases no enforcement at all and in both cases no significant threat is posed to countries’ autonomy and sovereignty. Shortly we will come to the issue of enforcement.

Therefore, with specific regards to justice in global health particularly health financing, in order to fulfil the health rights of marginalised populations what needs to be done is to specify the minimum obligations for each set of actors; that is, donor and recipient countries. Our discussion has been intended to demonstrate that this specificity and potential enforcement are critical requirements if a certain minimum is to be guaranteed. The task of specifying these obligations requires first of all establishing the total cost of ensuring a minimum distribution of health goods and services in each country (to which all individuals should have a right) and ultimately the total cost for all countries. It is this total cost that should be equitably shared by the two sets of actors by specifying exactly what portions external and domestic governments are jointly morally required to contribute in order to cover the cost of the minimum health opportunities which constitute the basic health rights of marginalised populations [24]. After this step, each set of actors should find a mechanism for equitably sharing their quota of the burden, taking into consideration differences in resource capacities of different actors within each set of actors.

This can be briefly illustrated. Even though the WHO provides an estimate of US$ 44 as the minimum health expenditure per capita needed by citizens of LICs to achieve the initial minimum or just health opportunities (basic life-saving services), so far there is no suggestion as to how the burden of raising this amount for each individual should be divided between LIC and HIC governments. We have proposed and elaborated such a mechanism elsewhere [24]. Once such specification has been made in a manner that is acceptable as equitable between HIC and LIC governments, it is reasonable to expect that each party will fully comply with its obligations without coercion or with the most minimum coercion that leaves states’ sovereignty and autonomy unthreatened. But even in the absence of enforcement, it will moreover be possible to objectively identify and assign moral blame to States Parties that flout their very specific obligations. Once the initial minimum of health opportunities or health rights has been achieved in this manner, more incremental obligations can be agreed upon and implemented in a similar manner. Therefore, using the idea of “a minimal conception of justice” which yields potentially less demanding obligations, especially extraterritorial obligations, along with “a thin system” of global enforcement, it is possible to significantly reduce global health injustice by being able to fulfil the basic health rights of marginalised populations of LICs in an incremental manner while bypassing the deeply controversial theoretical and technical difficulties.

Regarding enforcement of countries’ obligations, our discussion has shown that all fundamental reasons for the futility of current obligations have something to do with fear of potential erosion of national sovereignty and autonomy. However, in her response to the unwillingness to specify and enforce obligations of global distributive justice stemming from a desire to maintain national autonomy and sovereignty, Martha Nussbaum has convincingly argued that “[…] there is not any reason why a thin system of global governance, with at least some coercive powers, should not be compatible with the sovereignty and freedom of individual nations” (emphasis added) [6]. However, in her view the enforcement is supposed to apply to a broader spectrum of all relevant actions as may be required for the achievement of global justice in general, rather than immediate redistribution of resources or health resources in particular. She espouses a very simple, democratic, and yet potentially feasible model of enforcement like one suggested by Thomas Pogge for enforcing his Global Resource Dividend (GRD) proposal [46]. In respect of this view it ought to be added that not only is it possible to enforce obligations of global distributive justice without deeply jeopardising national sovereignty and autonomy, it is also a requirement if we are aiming at guaranteeing a certain threshold of health opportunities to all marginalised populations and individuals. The evidence for the feasibility of this enforcement can be found in the existence and demonstrated successful operation of regional blocs such as the European Union (EU); the Economic Organisation of West African Countries (ECOWAS) among others. In all these arrangements there is an acceptable degree of constraint on national autonomy and sovereignty. Therefore, it is possible to enforce certain minimum measures that do not ask too much in terms of countries’ autonomy and sovereignty. In this regard our view is that Nussbaum’s concept of a minimal conception of social justice, along with her proposal of a thin global enforcement system are consistent with the political values of national autonomy and sovereignty.

Lastly it is crucial to caution that in order to guarantee the legitimacy of these obligations and their enforcement, the relevant obligations and related decisions cannot be arrived at arbitrarily and simply imposed on States Parties. The process of determining specific obligations of each country or State Party has to be democratic (involving country representatives); evidence-based especially regarding the optimum resource-capacities of different countries; multidisciplinary in nature involving economists; health economists; human rights lawyers; professional ethicists and all relevant experts.

## Conclusion

Current obligations of countries for global distributive justice have been largely futile and this explains why it has remained difficult for LIC citizens to realise their basic health rights. This futility affects both domestic and extraterritorial obligations. At a superficial level the futility of countries’ obligations for global distributive justice can be attributed to the vagueness and lack of rigour that characterise them as well as their lack of enforcement. However, a deeper examination of these reasons reveals that the weaknesses within these obligations are not spontaneous. They are due to lack of political will to specify and enforce domestic and transnational obligations of social or particularly distributive justice. Further, reluctance to propose and implement an enforcement mechanism for these obligations is due to pessimism about the desirability and feasibility of a global enforcement system which is feared to erode countries’ national sovereignty and autonomy, and at worst the threat of imperialism. However, in the current global political circumstances there is a lot of evidence that there can be “a thin” and yet effective system of enforcement which is consistent with countries’ rights to national autonomy and sovereignty. Given the “minimal conception of social justice” we should agree on less-demanding obligations which guarantee, for example, a certain minimum amount of health goods and services (expressed as minimum health expenditure per capita per country) to all marginalised populations. We envisage that such obligations will be easily fulfilled with minimum or no enforcement at all. With regards to health financing, since it is possible to know the required minimum health expenditure per capita of each citizen in LICs, the obligation to meet these costs should be equitably apportioned between LIC and HIC governments by specifying exactly what portion each actor should contribute, taking into account the optimal resource capacity of each set of actors and individual actors within each set. Other obligations, especially of LIC governments should also be specified which, if fulfilled, would ensure efficient and equitable health systems domestically and globally. Such a strategy will go a long way towards fulfilment of the health rights of LIC citizens and all marginalised populations.

## Abbreviations

DAC, Development Assistance Committee; ESCR, Economic Social and Cultural Rights; GDP, Gross Domestic Product; GNI, Gross National Income; GRD, Global Resource Dividend; HIC, High Income Country; ICESCR, International Covenant on Economic Social and Cultural Rights; IMF, International Monetary Fund; LIC, Low Income Country; LMIC, Low and Middle Income Country; MDGs, Millennium Development Goals; ODA, Official Development Assistance; WHO, World Health Organisation; WTO, World Trade Organisation.

## Footnotes

1 In this case proportionality (fairness) of the burden takes into the optimal resources available to each set of actors (i.e., HIC Vs LICs). Our view is that once it can be demonstrated that LICs have already borne the maximum burden they can by using the maximum of their available resources to fulfill their citizens’ health rights, then it is most likely that HIC governments will be morally compelled to fulfill their quota of obligation or there will be a strong moral justification for the enforcement of such obligations upon recalcitrant states.

2 Nussbaum’s categorical disregard for “the level of opulence” of a society can be controversial if it is stated, as it is, without further qualification. This is especially so if she means that to qualify as just all societies must, with or without foreign assistance; ensure a certain threshold level of material and non-material entitlements to all citizens. Levels of poverty (at a national level) in most resource-poor countries may make it difficult to achieve a certain minimum level of material well-being without external assistance even though their internal arrangements are just. In such a case it might be problematic to judge such a society as unjust for the mere reason that they cannot afford the threshold for all their citizens.
